# Integrative Identification of Hub Genes Associated With Immune Cells in Atrial Fibrillation Using Weighted Gene Correlation Network Analysis

**DOI:** 10.3389/fcvm.2020.631775

**Published:** 2021-01-21

**Authors:** Tao Yan, Shijie Zhu, Miao Zhu, Chunsheng Wang, Changfa Guo

**Affiliations:** Department of Cardiovascular Surgery, Zhongshan Hospital, Fudan University, Shanghai, China

**Keywords:** atrial fibrillation, WGCNA, immune cells, bioinformatics, hub genes

## Abstract

**Background:** Atrial fibrillation (AF) is the most common tachyarrhythmia in the clinic, leading to high morbidity and mortality. Although many studies on AF have been conducted, the molecular mechanism of AF has not been fully elucidated. This study was designed to explore the molecular mechanism of AF using integrative bioinformatics analysis and provide new insights into the pathophysiology of AF.

**Methods:** The GSE115574 dataset was downloaded, and Cibersort was applied to estimate the relative expression of 22 kinds of immune cells. Differentially expressed genes (DEGs) were identified through the limma package in R language. Weighted gene correlation network analysis (WGCNA) was performed to cluster DEGs into different modules and explore relationships between modules and immune cell types. Functional enrichment analysis was performed on DEGs in the significant module, and hub genes were identified based on the protein-protein interaction (PPI) network. Hub genes were then verified using quantitative real-time polymerase chain reaction (qRT-PCR).

**Results:** A total of 2,350 DEGs were identified and clustered into eleven modules using WGCNA. The magenta module with 246 genes was identified as the key module associated with M1 macrophages with the highest correlation coefficient. Three hub genes (*CTSS, CSF2RB*, and *NCF2*) were identified. The results verified using three other datasets and qRT-PCR demonstrated that the expression levels of these three genes in patients with AF were significantly higher than those in patients with SR, which were consistent with the bioinformatic analysis.

**Conclusion:** Three novel genes identified using comprehensive bioinformatics analysis may play crucial roles in the pathophysiological mechanism in AF, which provide potential therapeutic targets and new insights into the treatment and early detection of AF.

## Introduction

Atrial fibrillation, the most common sustained arrhythmias in the clinic (1), affects ~1 to 2% of the population (2), which increases the morbidity, mortality, and medical burden worldwide (3). The risk of stroke in patients with AF is approximately five-fold higher than that in healthy people (4). AF is a complex disease (5) associated with common risk factors, including advancing age, male sex, hypertension, obesity, and diabetes (6). However, the pathophysiological mechanism leading to AF is still unclear. Increasing evidence demonstrates that immune cells play a significant role in the pathogenesis of AF (7). Several immune-mediated serum inflammatory markers such as *CRP* and *IL-6* have been confirmed to be elevated in patients with AF (8, 9). Nevertheless, the association between immune cells and the biological molecular mechanism of AF still needs further research to clarify the pathogenesis of AF and find potential therapeutic targets.

Weighted gene correlation network analysis (WGCNA) is a system biology method used to cluster genes into modules according to expression patterns among different samples (10). Based on the interconnectivity of genes, WGCNA can explore the relationships between gene modules and the clinical phenotypes to identify candidate biomarkers or therapeutic targets. It has been applied to numerous kinds of diseases (11–14).

In this study, we aimed to explore the association between immune cells and AF using comprehensive bioinformatics analysis. Cibersort was used to evaluate immune cell composition, and WGCNA was used to identify the hub gene module. We then analyzed the functional enrichment of the genes in the hub module. Based on the protein-protein interaction (PPI) network, hub genes were identified for further analysis and validation. We hope that this research can provide potential targets and new research ideas for the treatment of AF.

## Methods and Materials

### Data Acquisition and Processing

The GSE115574 dataset, including fourteen gene expression profiles from left atrial tissues of AF patients and fifteen gene expression profiles from left atrial tissues of sinus rhythm (SR) patients, were downloaded in the Gene Expression Omnibus (GEO) database. The R language was used to process the original expression profile of the GSE115574 dataset. Cibersort, a bioinformatics algorithm that could estimate 22 immune cell types, was applied to evaluate immune cell composition based on the gene expression matrix (15). The Linear Models for Microarray data (limma) package in the R language (16) was utilized to identify differentially expressed genes (DEGs) with a *p* < 0.05 between patients with AF and SR. The GSE31821, GSE41177, and GSE79768 datasets were used for validation. The batch effect was removed using the SVA package in the R language.

### WGCNA

The WGCNA (10) was applied to construct the mRNA co-expression network based on the DEGs. Briefly, an appropriate soft-thresholding power β was determined to realize the scale-free topology. Then DEGs were clustered into modules, which were labeled with different colors using the average linkage hierarchical clustering method. The minimum number of genes in each module was twenty, and the threshold for module merging was 0.25. Pearson's correlation method was utilized to calculate the correlation between each module and the relative expression of immune cells identified by the Cibersort. The module with the highest correlation coefficient was selected for further analyses.

### Functional Enrichment Analyses

The Database for Annotation, Visualization and Integrated Discovery (DAVID, v6.8) (17) was used to perform the Gene Ontology (GO) (18, 19) and Kyoto Encyclopedia of Genes and Genomes (KEGG) pathway enrichment analyses, which revealed the biological processes (BPs), cellular components (CCs), molecular functions (MFs), and pathways related to genes in the module identified above. GO terms and KEGG maps with a *p* < 0.05 were considered significant enrichment.

### Construction of PPI Network and Hub Genes Identification

The DEGs in the selected module were imported into the Search Tool for the Retrieval of Interacting Genes (STRING, v11.0) (20) to generate the PPI network identifying the interactions between the genes with the threshold of interaction score >0.4. Nodes represent proteins, and edges represent protein-protein associations in the PPI network. The results downloaded from the STRING database was then visualized utilizing Cytoscape software (v3.8.1). CytoHubba (21), a plug-in of the Cytoscape software, was used to identified hub genes. The intersection of five algorithms in CytoHubba was generated for hub genes identification to ensure the accuracy and robustness of the results.

### Sample Collection

Adult patients with persistent AF undergoing cardiac surgery in Zhongshan Hospital were included in this study. Persistent AF is defined as AF which is continuously sustained beyond 7 days, including episodes terminated by cardioversion (drugs or electrical cardioversion) after more than seven days (3). Excluded from the research were patients with coronary artery heart disease, hypertension, diabetes, or obstructive sleep apnea syndrome, whose ejection fractions were <30%, and those who had contraindications to surgery. The left atrium tissues and blood samples of twenty patients with persistent AF and ten healthy donors with SR were collected during cardiac surgery. The samples were then immediately preserved in liquid nitrogen for the later experiment. This study was in full compliance with the Declaration of Helsinki and approved by the Medical Ethics Committee of Zhongshan Hospital, Fudan University (Approval No. B2019-198R). All patients participating in this study have signed written informed consent before surgery.

### Quantitative Real-Time Polymerase Chain Reaction (qRT-PCR)

Total left atrium tissue RNA was extracted with the RNeasy™ Mini Kit (QIAGEN, Frankfurt, Germany) following the manufacturer's instruction. The PrimeScript™ RT reagent Kit (Takara, Otsu, Japan) was used to conduct reverse transcription. QRT-PCR was performed with the TB Green^®^ Premix Ex Taq™ II (Takara, Otsu, Japan) on QuantStudio™ 5 System (Thermo Fisher Science, Waltham, MA, USA). The expression data was normalized by GAPDH, and the 2^−ΔΔCT^ method was applied to analyze the results. All sequences for RNA primers (Sangon Biotech, Shanghai, China) are shown in Table 1.

**Table 1:** Lists of primer sequences used for quantitative real-time PCR.

**Genes**	**Sequences**
*GAPDH*	Forward: GGAGCGAGATCCCTCCAAAAT Reverse: GGCTGTTGTCATACTTCTCATGG
*CTSS*	Forward: TGACAACGGCTTTCCAGTACA Reverse: GGCAGCACGATATTTTGAGTCAT
*CSF2RB*	Forward: CTCCTTTGGCCTATTCTACAAGC Reverse: TGAACAGAGACGATGTATTGGC
*NCF2*	Forward: CCAGAAGCATTAACCGAGACAA Reverse: CCTCGAAGCTGAATCAAGGC

## Results

### Identification of DEGs

A total of 2,350 genes, including 1,115 upregulated and 1,235 downregulated, which were differentially expressed between AF samples and SR samples, were identified in the GSE115574 dataset with the limma package.

### Relative Immune Cells Expression

**Figure 1:**
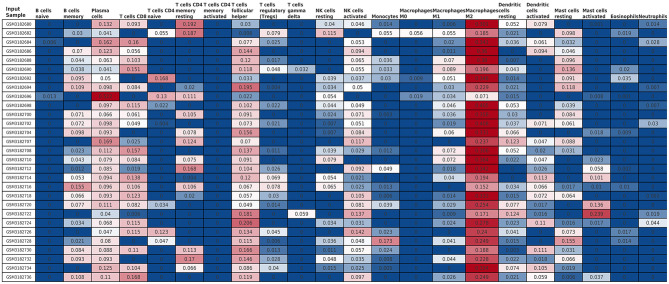
The relative expression of 22 immune cell subtypes in each sample estimated using Cibersort. The relative expression was higher from blue to red.

**Figure 2:**
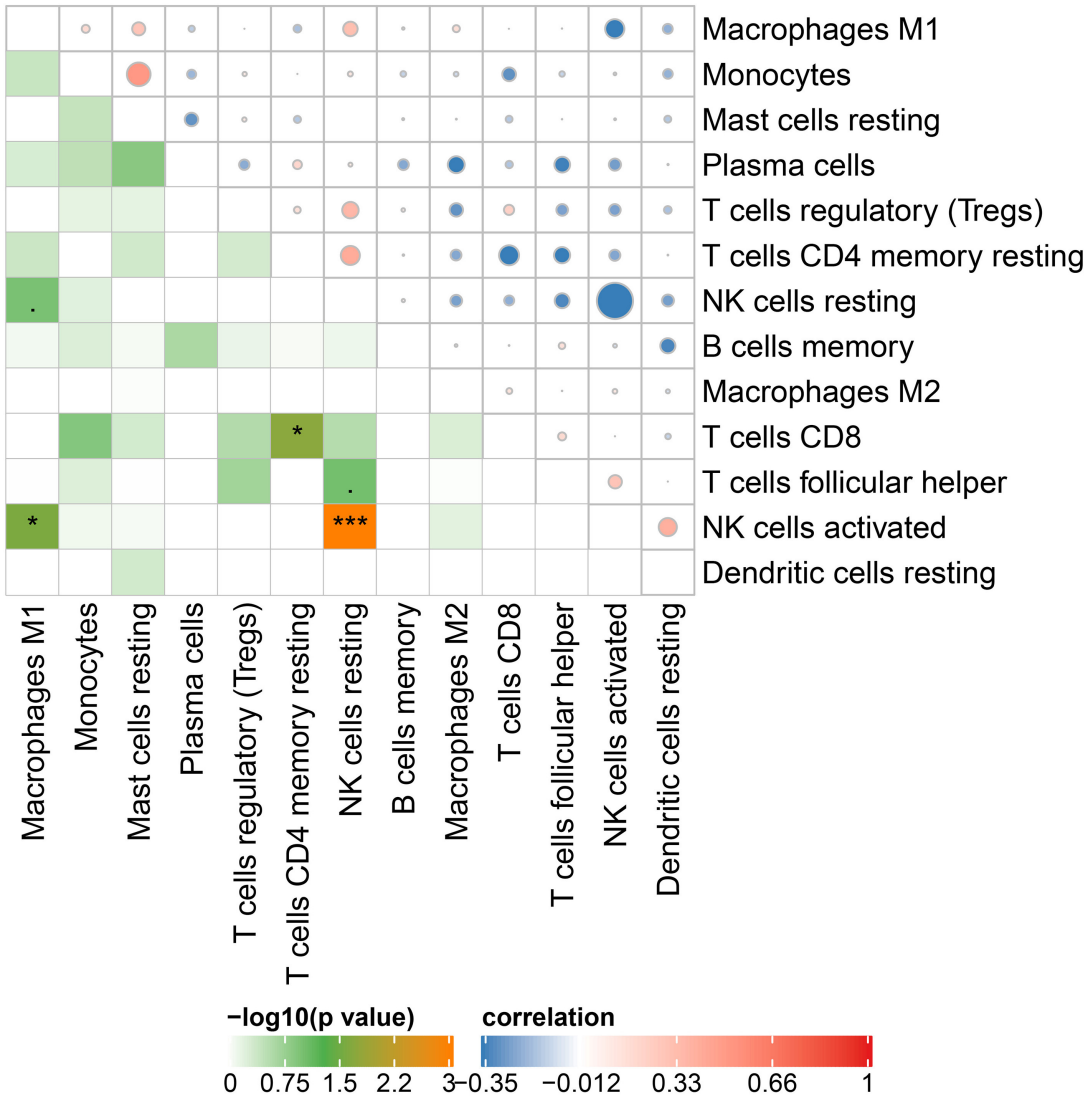
Correlation matrix of 13 immune cell subtype compositions. Blue dots represent negative correlation, and red dots represent positive correlation. The size of the dot is positively correlated with the correlation coefficient. ^*^p < 0.05 and ^***^p < 0.001.

The Cibersort with 22 types of immune cell subtypes was applied to estimate the putative relative expression of immune cells. The results are shown in Figure 1. Naïve B cells, CD4 naïve T cells, CD4 memory activated T cells, gamma delta T cells, resting NK cells, M0 macrophages, activated mast cells, eosinophils, and neutrophils were eliminated because most of the samples were not inferred to express in these immune cells. The correlation of remaining immune cells was calculated using the Pearson correlation coefficient, as shown in Figure 2. M1 macrophages were significantly negatively correlated with activated NK cells (*r* = −0.42, *p* = 0.0247). CD4 memory resting T cells were significantly negatively correlated with CD8 T cells (*r* = −0.43, *p* = 0.0191). Resting NK cells were significantly negatively correlated with activated NK cells (*r* = −0.78, *p* < 0.001).

### Construction of the Weighted Coexpression Networks

**Figure 3:**
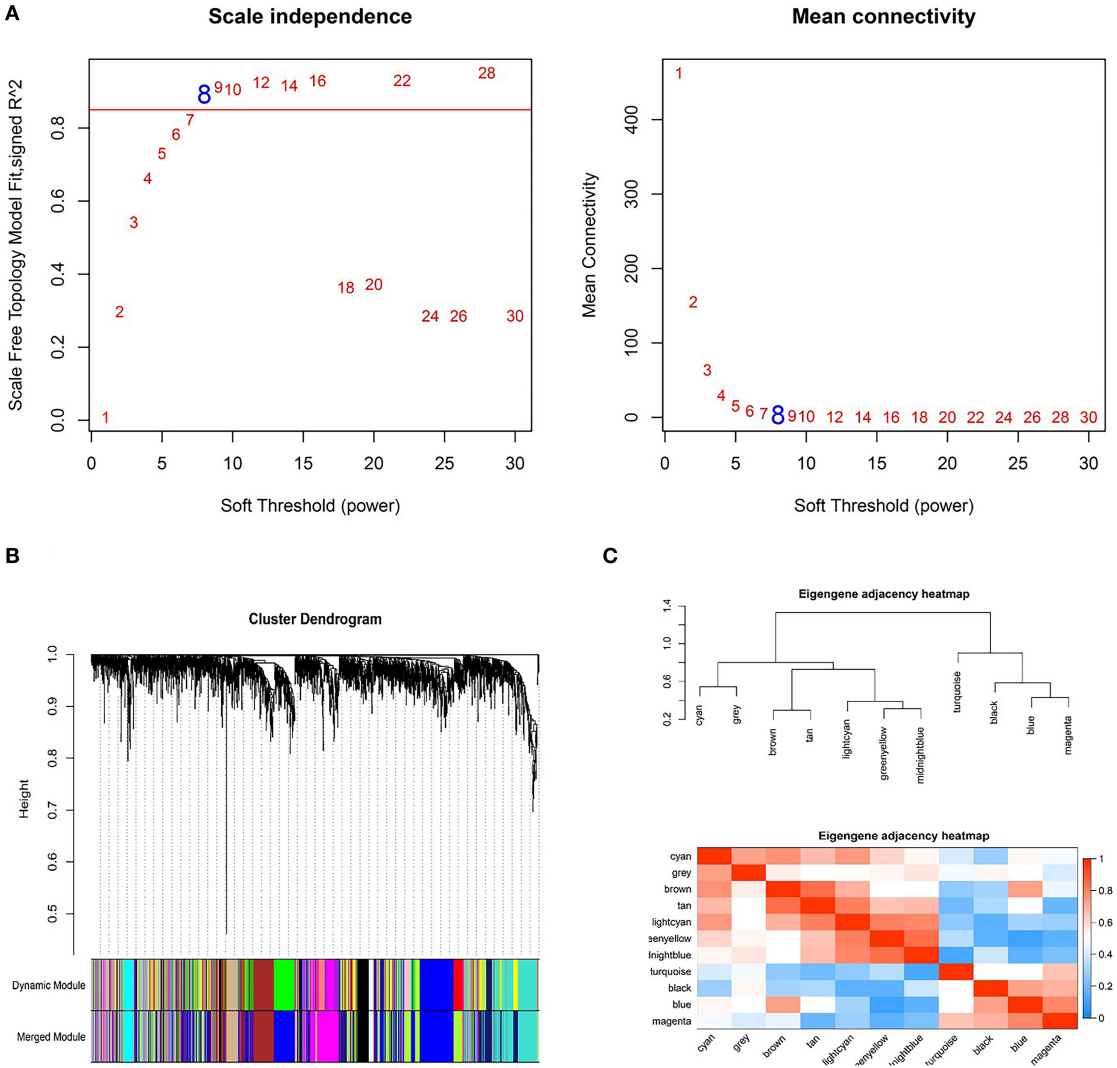
Weighted genes correlation network analysis to cluster genes into different modules. **(A)** The selection of the soft-thresholding power β. **(B)** Dendrogram of all differentially expressed genes. **(C)** The clustering heat map between modules. Red means closer similarity, and blue means farther similarity.

Based on the DEGs identified above, a total of 2,350 genes were subjected to WGCNA. We then established a scale-free (scale-free *R*^2^ > 0.85) coexpression network with the soft-thresholding power β = 8. Using the average linkage hierarchical clustering method, DEGs were clustered into eleven modules with different colors, including black, blue, brown, cyan, greenyellow, gray, lightcyan, magenta, midnightblue, tan, and turquoise (Figure 3).

### Correlation Between Modules and Immune Cell Types

**Figure 4:**
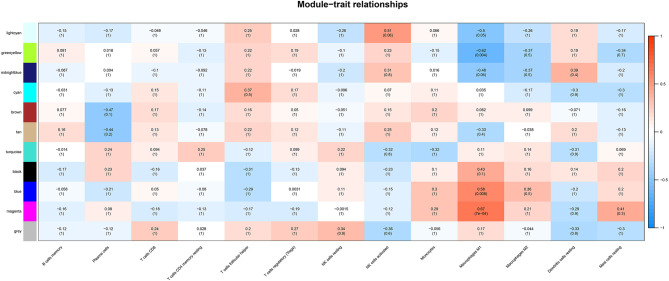
The heatmap showing module-trait correlations. Blue represent negative correlation, and red represent positive correlation. The magenta module had the strongest correlation with M1 macrophages.

Correlation analysis was performed between each module and immune cell types selected above, including memory B cells, plasma cells, CD8 T cells, CD4 memory T cells, follicular helper T cells, regulatory T cells (Tregs), resting NK cells, activated NK cells, monocytes, M1 macrophages, M2 macrophages, resting dendritic cells, resting mast cells. The results demonstrated that the magenta module (*r* = 0.67, *p* < 0.001) and the blue module (*r* = 0.58, *p* = 0.008) were significantly positively correlated with M1 macrophages, while the greenyellow module (*r* = −0.62, *p* = 0.004) was significantly negatively correlated with M1 macrophages, as shown in Figure 4. The magenta module with 246 genes was identified as the key module associated with M1 macrophages with the highest correlation coefficient.

### Functional Enrichment Analyses

**Figure 5:**
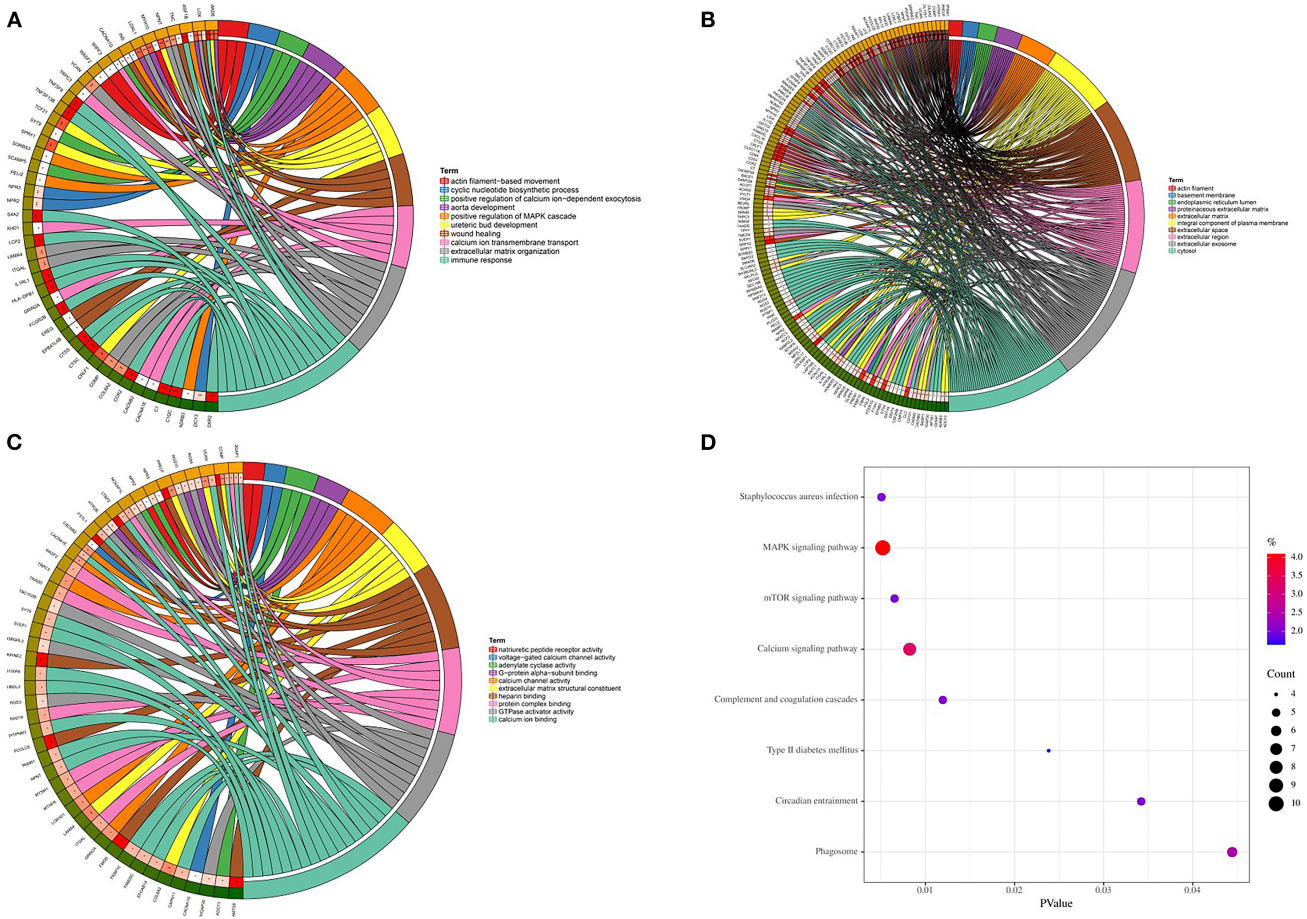
GO and KEGG enrichment analyses. **(A)** Biological process. **(B)** Cellular component. **(C)** Molecular function. **(D)** KEGG pathways.

Genes in the magenta module were selected to perform GO and KEGG functional enrichment analyses utilizing the DAVID online tool to investigate the biological effects, as shown in Figure 5. The significant enriched BPs included immune response, ureteric bud development, aorta development, extracellular matrix organization, cyclic nucleotide biosynthetic process, and calcium ion transmembrane transport. In addition, extracellular matrix, extracellular space, extracellular region, basement membrane, and actin filament were significant enriched in CC. For MF, the most significant entries were heparin binding, extracellular matrix structural constituent, calcium channel activity, protein complex binding, and calcium ion binding. Furthermore, the KEGG pathway analysis suggested that DEGs were mainly enriched in staphylococcus aureus infection, MAPK signaling pathway, mTOR signaling pathway, calcium signaling pathway, and complement and coagulation cascades.

### Construction of the PPI Network and Hub Genes Identification

**Figure 6:**
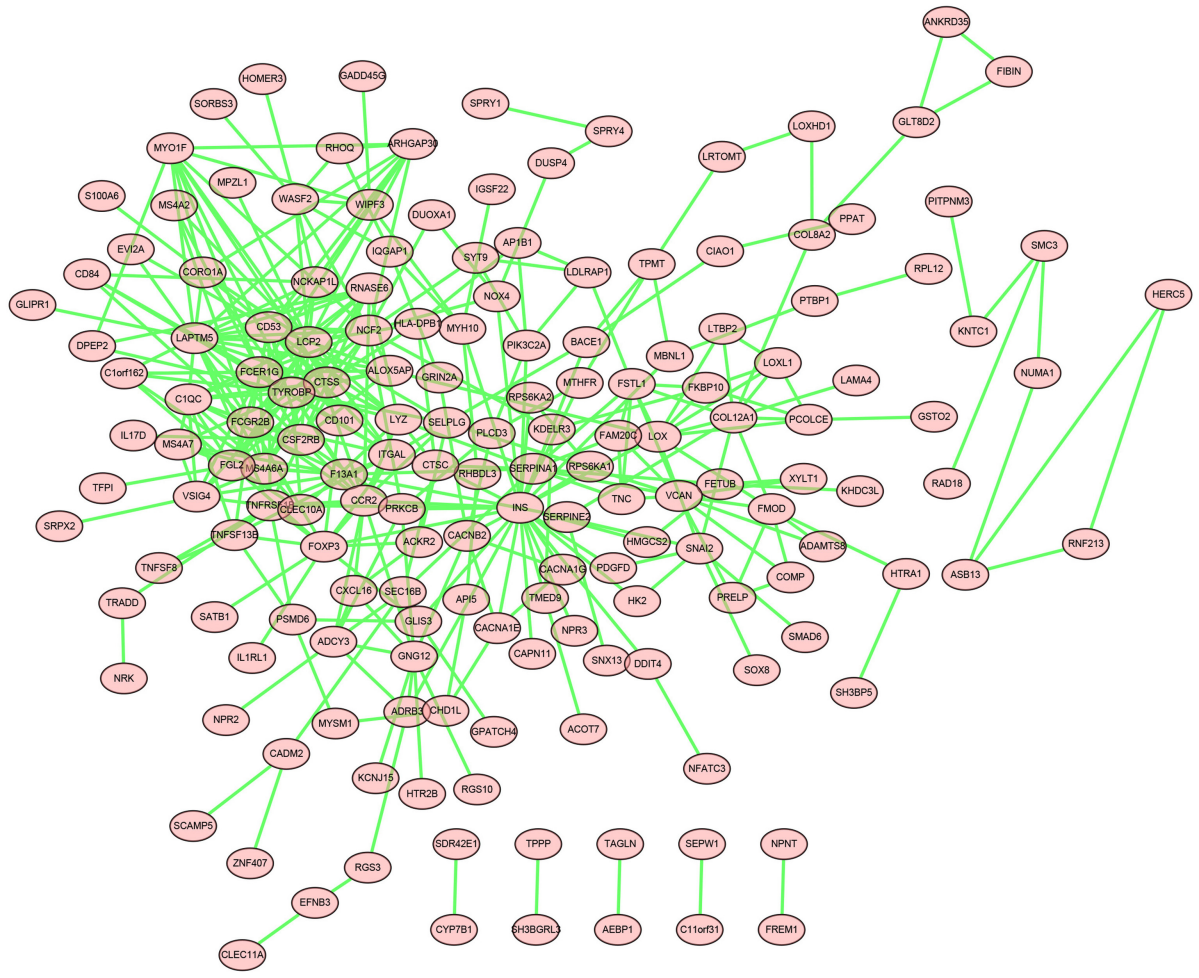
The PPI network of genes in the magenta module.

**Figure 7:**
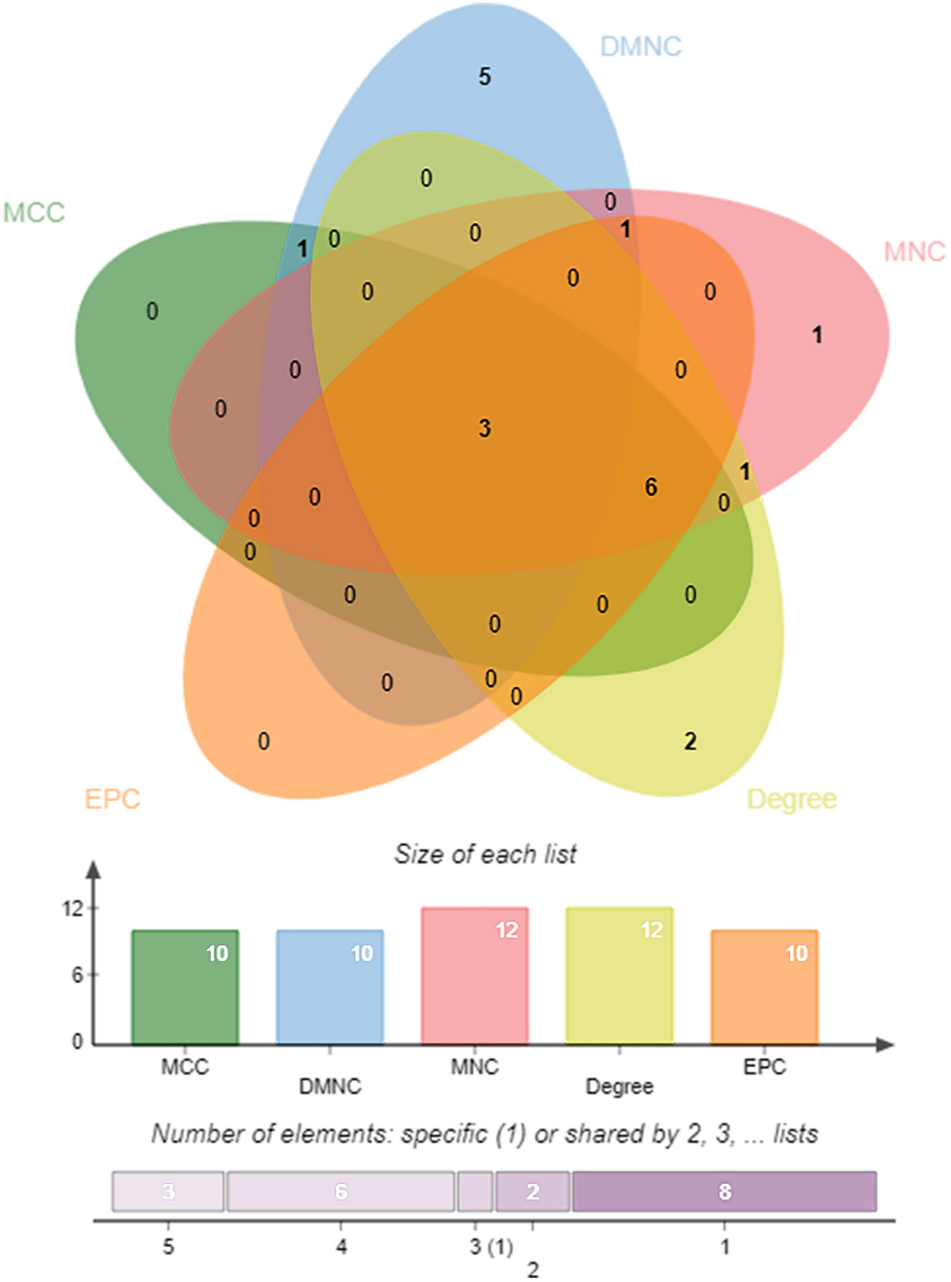
A Venn diagram between five algorithms of CytoHubba. The coincident part represents the three genes (*CTSS, CSF2RB*, and *NCF2*) identified by all five algorithms.

DEGs in the magenta module were imported into the STRING online tool to evaluate the interaction between these genes, and a total of 167 nodes and 393 edges were identified from the PPI network, as shown in Figure 6. Five algorithms of the CytoHubba, including MCC, DMNC, MNC, Degree, and EPC, were then applied to process the PPI network to identify the top ten genes, which are shown in Table 2. A Venn diagram (Figure 7) was generated to establish the intersection of genes identified by five algorithms, and *CTSS, CSF2RB*, and *NCF2* were determined as hub genes. These three genes may play considerable roles in the pathophysiology of AF.

**Table 2:** Top ten genes calculated by five algorithms of CytoHubba.

**Ranks**	**MCC**	**DMNC**	**MNC**	**Degree**	**EPC**
1	*TYROBP*	*ALOX5AP*	*TYROBP*	*INS*	*TYROBP*
2	*FCER1G*	*F13A1*	*LCP2*	*TYROBP*	*LCP2*
3	*CTSS*	*MS4A7*	*FCER1G*	*LCP2*	*LAPTM5*
4	*LCP2*	*VSIG4*	*LAPTM5*	*LAPTM5*	*FCER1G*
5	*CSF2RB*	*CTSS*	*FCGR2B*	*FCER1G*	*CSF2RB*
6	*LAPTM5*	*CORO1A*	*CD53*	*FCGR2B*	*CD53*
7	*CD53*	*ARHGAP30*	*CTSS*	*CD53*	*FCGR2B*
8	*NCF2*	*CSF2RB*	*CSF2RB*	*NCF2*	*CTSS*
9	*FCGR2B*	*NCF2*	*FGL2*	*CSF2RB*	*NCF2*
10	*ALOX5AP*	*C1QC*	*NCF2*	*CTSS*	*C1QC*

### Validation of the Hub Genes

**Figure 8:**
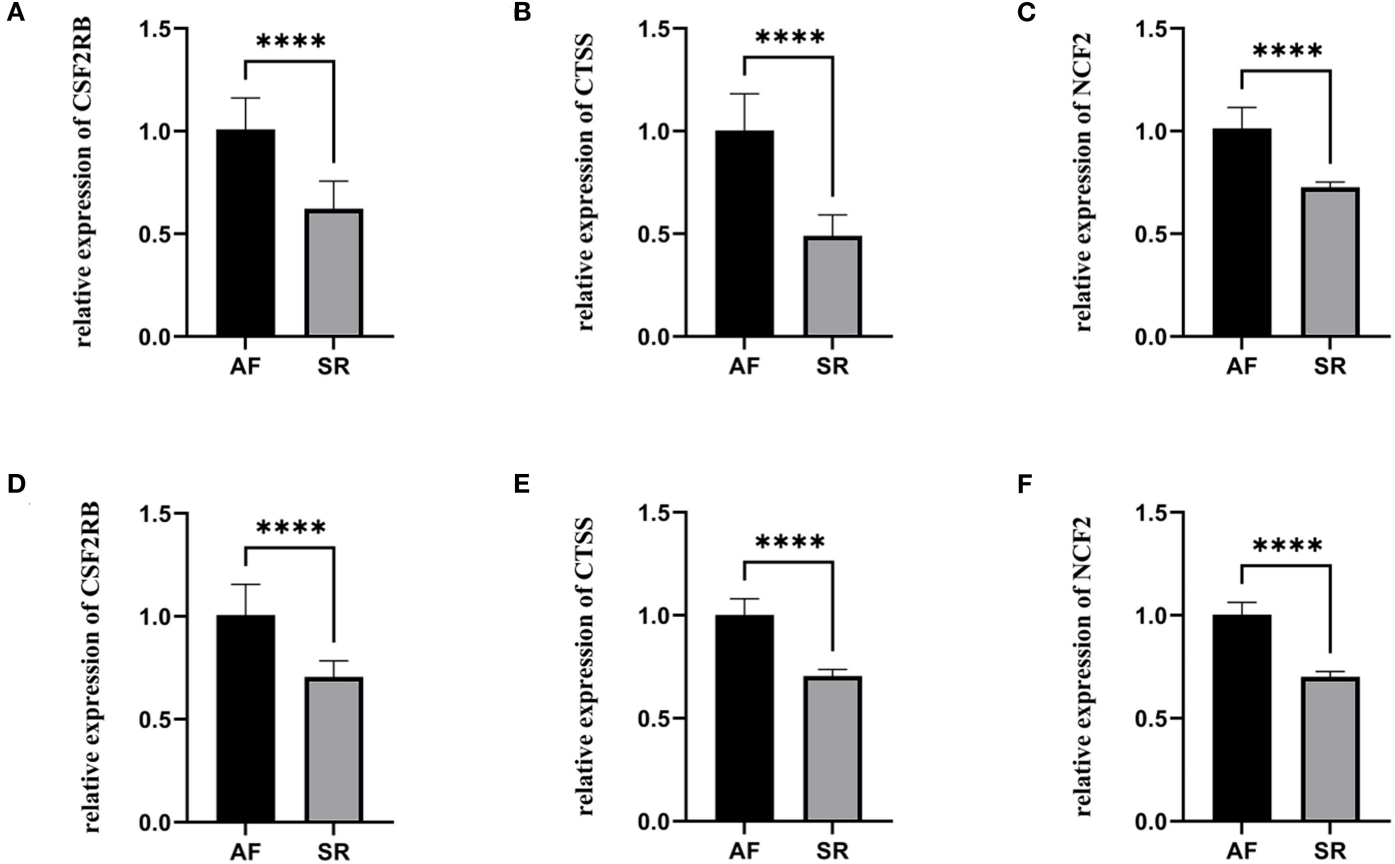
The relative expression of three hub genes between atrial fibrillation (AF) and sinus rhythm (SR). **(A–C)** The relative expression of *CSF2RB, CTSS*, and *NCF2* in left atriums. **(D–F)** The relative expression of *CSF2RB, CTSS*, and *NCF2* in blood samples. ^****^p < 0.0001.

**Figure 9:**
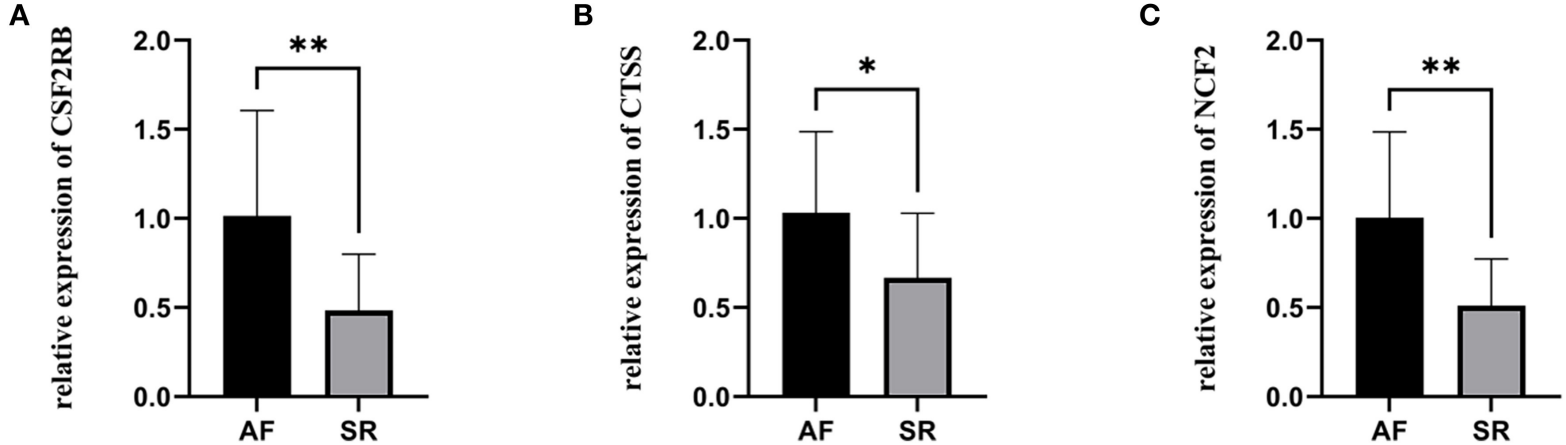
The relative expression of three hub genes verified by three datasets. **(A)** The relative expression of *CSF2RB*. **(B)** The relative expression of *CTSS*. **(C)** The relative expression of *NCF2*. ^*^p < 0.05, ^**^p < 0.01.

The expression levels of three hub genes were detected in LAs and blood samples, respectively, by qRT-PCR. The results showed that the expression levels of *CTSS, CSF2RB*, and *NCF2* in AF were significantly higher than those in SR both in LAs and blood samples, which were consistent with the bioinformatic analysis (Figure 8). Moreover, we combined three datasets, GSE31821, GSE41177, and GSE79768, to verify the expression levels of these three genes between AF and SR. The results also demonstrated that the expression levels of *CTSS, CSF2RB*, and *NCF2* were significantly higher in AF than those in SR (Figure 9).

## Discussion

AF is the most common tachyarrhythmia in the clinic. The typical clinical manifestations of AF are palpitations, fatigue, chest tightness, and decreased exercise tolerance, which seriously affect the life quality of patients (22). AF increases the risk of ischemic stroke by five times higher than healthy people and leads to high morbidity and mortality (23). Despite the fact that lots of efforts have been made, however, the molecular mechanism of AF development is still not completely understood. Therefore, it is significant urgent to clarify the pathogenesis of AF and find potential therapeutic targets.

In this study, we downloaded the GSE115574 dataset from the GEO database and estimated the composition of the immune cells using Cibersort based on the expression matrix. WGCNA was performed to determine the module with the most robust relationship between genes in the module and immune cell types. A total of eleven modules were identified, and the magenta module was significantly correlated with M1 macrophages. To the best of our knowledge, it is the first time that WGCNA has been used to analyze the relationships between immune cell types and AF. We then performed enrichment analysis on genes in the magenta module. According to GO analysis, genes were mainly enriched in immune response, ureteric bud development, aorta development, and extracellular matrix organization. KEGG pathway analysis demonstrated that genes were mainly enriched in staphylococcus aureus infection, MAPK signaling pathway, and mTOR signaling pathway. Based on the PPI network and CytoHubba, we identified three hub genes, including *CTSS, CSF2RB*, and *NCF2*. As we know, the relationships between these three genes and the molecular mechanism of AF has not been studied, which is worth further research. Finally, qRT-PCR was performed to verify the expression of *CTSS, CSF2RB*, and *NCF2*. The expression levels of these three genes in patients with AF were significantly higher than those in patients with SR, which were consistent with the bioinformatic analysis. The expression levels of these three genes were also verified in three other datasets, GSE31821, GSE41177, and GSE79768.

Previous studies have shown that macrophages are associated with atrial fibrosis, leading to structural remodeling in the process of AF (2, 7, 24). Cytokines released by macrophages such as tumor necrosis factor-α (*TNF-*α) and interleukin-1β (*IL-1*β) can activate fibroblast proliferation, leading to fibrous tissue formation (25). However, few studies have investigated the precise role of these cytokines in the molecular mechanism of AF.

*CTSS* is a lysosomal cysteine proteinase, playing a crucial role in the degradation of antigenic proteins on major histocompatibility complex (MHC) class II molecules (26). *CTSS* is associated with many inflammatory and autoimmune diseases. *CTSS* expressed by intimal macrophages was involved in atherogenesis, and deficiency of *CTSS* could reduce atherosclerosis in LDL receptor-deficient mice (27). *miR4498*/*CTSS* might polarize macrophages into pro-inflammatory phenotype and accelerate chronic atherosclerotic inflammation (28). *CTSS* participated in the abdominal aortic aneurysm (AAA) formation, and inhibition of *CTSS* suppressed AAA formation in mice (29). A previous study showed that *CTSS*-mediated induction of *CX3CL1* might contribute to the ocular surface and lacrimal glands inflammation in Sjögren's syndrome with a 4.5-fold increase in *CX3CR1*-expressing macrophages (30). In chronic obstructive pulmonary disease, reduction in *CTSS* expression prevents loss of lung function, reduces inflammation, and slows the lung tissue remodeling (31). Moreover, *CTSS* was identified as novel biomarkers for diseases and physiological processes, including triple-negative breast cancer, sarcoidosis, and particulate-induced lysosomal disruption in macrophages (32–34).

*CSF2RB* is the common beta chain of the high affinity receptor for interleukin-3, interleukin-5, and colony-stimulating factor. The role of *CSF2RB* has been studied in many diseases. Runt-related transcription factor 1 directly bound to the promoters of *CSF2RB*, which regulated apoptosis of neuroblastoma (35). The activating hotspot mutation in *CSFR2B* was identified in myeloid leukemia in Down syndrome (36). The mutation in *CSF2RB* can also cause hereditary pulmonary alveolar proteinosis (PAP) (37). In *CSF2RB*-/- mice, statin therapy reduces cholesterol accumulation in alveolar macrophages and ameliorates PAP (38). Moreover, *CSF2RB* was found overexpressed on monocytes from Alzheimer's disease patients, which contributed to granulocyte-macrophage colony-stimulating factor-induced monocyte migration (39). However, the role of *CSF2RB* has never been studied in AF. Further research is required to determine whether *CSF2RB* can become a novel therapeutic target for AF.

*NCF2*, encoding neutrophil cytosolic factor 2, mainly results in autoimmune diseases. In a previous case-control study, four single-nucleotide polymorphisms within the *NCF2* gene were genotyped, and the rs10911362 variants were associated with a decreased TB risk in the Western Chinese Han population (40). *NCF2* deficiency resulted in granulomas, and the *NCF2* mutation caused diverse and unusual clinical phenotype of chronic granulomatous disease (41, 42). In multiple sclerosis, *NCF2* was identified to be associated with eleven single nucleotide polymorphisms (43). Nevertheless, the relationship between *NCF2* and AF has not been elucidated.

There are some limitations to our study. First, the data we used was from public databases, which were limited in the sample size. Further prospective studies on more patients should be carried out to support our results. Second, although we have performed qRT-PCR to verify the expression levels of genes, mechanistic studies need to be conducted.

## Conclusion

In this study, we performed WGCNA to analyze the relationships between immune cell types and AF for the first time. Three novel genes (*CTSS, CSF2RB*, and *NCF2*) which have never been studied in AF were identified. These three genes are worthy of further study and may become potential therapeutic targets in AF.

## Data Availability Statement

The dataset presented in this study can be found at https://www.ncbi.nlm.nih.gov/geo/query/acc.cgi?acc=GSE115574, https://www.ncbi.nlm.nih.gov/geo/query/acc.cgi?acc=GSE31821, https://www.ncbi.nlm.nih.gov/geo/query/acc.cgi?acc=GSE41177, and https://www.ncbi.nlm.nih.gov/geo/query/acc.cgi?acc=GSE79768.

## Ethics Statement

The studies involving human participants were reviewed and approved by Medical Ethics Committee of Zhongshan Hospital, Fudan University. The patients/participants provided their written informed consent to participate in this study.

## Author Contributions

CG and CW conceived and designed this study. TY, SZ, and MZ contributed to data analysis and prepared the main manuscript. All authors reviewed the final manuscript.

## Conflict of Interest

The authors declare that the research was conducted in the absence of any commercial or financial relationships that could be construed as a potential conflict of interest.
